# The next phase in Long COVID research: addressing the ethical challenges in trials of disease-modifying treatments

**DOI:** 10.1016/j.eclinm.2026.103918

**Published:** 2026-05-07

**Authors:** Saskia Hendriks, Christine Grady, Megan L. Fitzgerald, Rachel S. Gross, Christine Maughan, Michael J. Peluso, Sumeeta Varma, Avindra Nath, Annette Rid

**Affiliations:** a Department of Bioethics, Clinical Center, National Institutes of Health, Bethesda, MD, USA; b Brain Inflammation Collaborative, Inc., Delafield, WI, USA; c Patient Led Research Collaborative, Calabasas, CA, USA; d Division of General Pediatrics, Department of Pediatrics, Department of Population Health, NYU Grossman School of Medicine, New York, USA; e Utah Covid-19 Long Haulers, Salt Lake City, UT, USA; f Division of HIV, Infectious Diseases, and Global Medicine, University of California, San Francisco, San Francisco, CA, USA; g Independent Scholar; h Section of Infections of the Nervous System, National Institute of Neurological Disorders and Stroke, National Institutes of Health, Bethesda, MD, USA; i Fogarty International Center, National Institutes of Health, Bethesda, MD, USA

**Keywords:** Long COVID, Treatment, Clinical trials, Research ethics, Post-acute COVID-19 syndrome, Ethics, Research

## Abstract

Almost five years after COVID-19 emerged, multiple scientific uncertainties remain about why some people experience ongoing symptoms long after being infected with SARS-CoV-2 (Long COVID). The pathophysiology underlying Long COVID and its potential to represent several endotypes are still under investigation. These scientific uncertainties around Long COVID have been cited as a reason to delay treatment trials until the disease is better understood. In this paper, a group of bioethicists, clinician-scientists and people with lived experience with Long COVID argue that it is ethically imperative to conduct trials of disease-modifying treatments for Long COVID now. Furthermore, we argue that although conducting such trials can pose ethical challenges, these challenges can be overcome through careful research priority-setting, rigorous trial design, fair participant selection, and ensuring that the risk-benefit profile is favorable.

## Introduction

An estimated 6–7% of adults and 2–20% of children report ongoing symptoms related to COVID-19 long after their initial illness.^1^, ^2^, ^3^ Long COVID, defined by the National Academies of Science, Engineering and Medicine (NASEM) as a disease state characterized by the presence of symptoms for at least 3 months after an acute SARS-CoV-2 infection,^4^ has major health, social and economic implications (Table 1).^7^^,^^13^^,^^29^, ^30^, ^31^, ^32^ Despite the significant disease burden,^13^, ^14^, ^15^, ^16^, ^17^ clinical care is currently limited to managing symptoms, improving function, and optimizing quality of life.^9^ While symptomatic management may be necessary, there is a need for disease-modifying treatments, defined as treatments that alter the course of the disease by targeting putative pathologic mechanisms of Long COVID, and hence go beyond relieving symptoms.^33^ Yet no disease-modifying treatments for Long COVID currently exist.^23^

**Table 1:** Current understanding of Long COVID and its impact.

Epidemiology	Although estimates vary widely (e.g., due to varying definitions of Long COVID), approximately 6–7% of adults and 2–20% of children infected with SARS-CoV-2 report ongoing symptoms long after the initial infection.^1^, ^2^, ^3^ In the US alone, ∼20 million adults and up to 5.8 million children are currently estimated to have Long COVID.^3^^,^^5^^,^^i^ At least 65 million individuals worldwide are estimated to have Long COVID.^6^
Clinical presentation, diagnosis, and pathogenesis	Some common symptoms of Long COVID in adults include fatigue, brain fog, dizziness, gastrointestinal symptoms, palpitations and post-exertional malaise.^7^^,^^8^ Currently, it is not possible to clearly exclude any particular symptom from representing Long COVID. Multiple different phenotypes are framed under the umbrella of Long COVID, yet whether these are all caused by SARS-CoV-2 is uncertain. Long COVID’s temporal pattern is variable: symptoms can be continuous, relapsing and remitting, progressive, improve, or resolve.^4^^,^^9^ The long-term prognosis is unclear.^6^ Most Long COVID research includes adults; the extent to which adult findings translate to children is underexplored. School-aged children, for example, have different symptoms than adolescents and adults.^10^ As there is no diagnostic test, Long COVID is a clinical diagnosis made in persons with symptoms that developed during or after probable or confirmed SARS-CoV-2 infection.^11^ There likely is considerable heterogeneity in who is diagnosed with Long COVID, considering that several definitions for Long COVID have been proposed, the definitions differ (e.g., as to whether they require the exclusion of other diagnosable conditions),^12^ and many definitions rely on clinical judgment to some extent.^12^
Disease burden	Long COVID’s disease burden is significant.^13^, ^14^, ^15^, ^16^, ^17^, ^18^ Around 80% of US adults with Long COVID experience activity limitations, and for 25% of US adults with Long COVID, these limitations are significant.^5^ For example, many people with Long COVID require a reduced work schedule or are not working due to the disease.^7^ Pediatric Long COVID can affect daily functioning, developmental milestones, and academic performance, although long-term impacts are still uncertain.^19^, ^20^, ^21^, ^22^
Disease management	Current disease management is based on knowledge about treating associated symptoms (e.g., Postural Orthostatic Tachycardia Syndrome) and is geared towards managing symptoms, improving function, and optimizing quality of life.^9^ There currently are no disease-modifying treatments.^23^
Affected populations	While anyone can get Long COVID, some groups are disproportionally affected (e.g., women).^9^^,^^22^ Insights into the role of social determinants of health are still limited,^24^^,^^25^ although difficulties in accessing care appear to disproportionately affect underserved populations.^9^^,^^22^
Societal impact	Long COVID’s disease burden likely has a major societal and economic impact (e.g., healthcare costs, effects on the labor force, educational attainment,^26^ caregiving burden^27^).^7^^,^^13^^,^^28^, ^29^, ^30^, ^31^, ^32^ The estimated annual global economic toll of Long COVID is estimated to be around USD 135 billion over the next decade.^28^

The development of such treatments is widely regarded as urgent,^6^^,^^31^^,^^33^, ^34^, ^35^, ^36^, ^37^, ^38^, ^39^, ^40^ and endorsed by the U.S. government and the World Health Organization.^9^^,^^41^ However, of the 339 therapeutic clinical trials on Long COVID registered on ClinicalTrials.gov as of September 2025, we estimate that only 78 (23%) tested treatments that may be considered disease-modifying. Of note, the uncertain pathophysiology of Long COVID makes it challenging to judge whether certain interventions have the potential to be disease modifying; we opted for an inclusive approach based on adjudication by two clinicians. Of the 78 listed trials, 24 were open for recruitment, 23 were not or not yet recruiting, 30 were completed and 1 had been terminated (Appendix 1,2). These trials tested immunomodulators (n = 31 trials), devices (n = 19), antiviral drugs (n = 14 trials), and miscellaneous agents (n = 19 trials), with some trials testing more than one modality. A significantly larger number of trials are needed with novel targets to expedite the development of disease-modifying treatments for Long COVID. But this raises an ethically significant question: when is the right time to conduct these trials?

The complex, heterogeneous and incompletely understood nature of Long COVID^4^^,^^33^^,^^35^ makes it currently difficult to decide what treatment candidates are most promising and creates significant scientific and ethical challenges in designing and conducting trials. For instance, studies have identified various potential therapeutic targets based on the putative pathophysiology of Long COVID, including antivirals for SARS-CoV-2 persistence, immunomodulators for autoimmunity and inflammation and anti-platelet drugs for thrombosis.^33^^,^^38^ However, because the given pathophysiological mechanisms have not been demonstrated in all people with Long COVID (PWLC) and standardized biomarkers for monitoring these mechanisms do not yet exist, selecting treatment candidates for testing, defining eligible populations, and developing ethically appropriate trial designs is riddled with uncertainty at this time. Conversely, if we postpone the pursuit of treatment trials and focus efforts on increasing our understanding of Long COVID until the mechanisms of disease are fully elucidated and biomarkers have been developed, there would be less uncertainty when starting trials of disease-modifying treatments, and each trial would be more likely to succeed. But millions of people who currently suffer from Long COVID would go longer without clinical trial opportunities and proven effective treatments that can alter the disease course.

In this paper, a group of bioethicists, clinician-scientists and PWLC argue that it is ethically imperative and feasible to conduct more trials of disease-modifying trials now. We first explain why more clinical trials of disease-modifying therapies are needed now. We then discuss foreseeable but underexplored ethical challenges in conducting such trials, given the specific constellation of challenges for Long COVID trials: the urgency of developing disease-modifying treatments, current scientific uncertainties around Long COVID, and disagreements about how to move the field forward. We use this analysis to make concrete suggestions for how to address these challenges, notably through careful research priority-setting, rigorous trial design, collaboration among researchers, industry, and regulators, and partnership with PWLC. Overall, we chart an ethical path for accelerating the development of disease-modifying treatments for Long COVID now.

Our discussion is the first to bridge literature on Long COVID and research ethics. To date, ethical analysis of clinical trials in long COVID has been limited; while a number of papers have made recommendations on how long COVID research should be conducted (e.g.,^19^^,^^36^^,^^40^^,^^42^^,^^43^), a rapid literature review showed only two take a dedicated ethical perspective^44^^,^^45^ (Appendix 3,4). Moreover, these dedicated ethical papers address specific issues: fair subject selection or inclusive research, they do not provide a systematic analysis. Specifically, we clarify and expand the ethical underpinnings of prior advocacy to conduct more clinical trials of disease-modifying treatments for Long COVID.^6^^,^^31^^,^^33^, ^34^, ^35^, ^36^, ^37^, ^38^, ^39^, ^40^ Additionally, we combine relevant literature to offer actionable ethical guidance for funders, sponsors, investigators, regulators, institutional review boards (IRBs), PWLC and others who are considering such trials. For example, we highlight neglected ethical topics in discussions about Long COVID (e.g., fair participant selection in high-demand trials^46^), adapt recent ethical recommendations to the specifics of disease-modifying treatment trials (e.g., regarding research priority-setting^47^ and fair participant selection^48^) and take clear positions on several ethically contested issues (e.g., use of placebo controls^49^). We thereby provide a valuable resource for those wishing to implement these trials. Finally, we believe our discussion can inform decisions about when and how to pivot from foundational observational research to clinical trials for other conditions that face similar challenges related to urgency, uncertainty and disagreement (e.g., emerging infectious diseases, post-acute infection syndromes and other incompletely understood conditions).

Throughout, we focus on clinical trials that assign participants to one or more disease-modifying treatments for Long COVID, or placebo or no treatment, to evaluate their clinical outcomes (sometimes abbreviated as “treatment trials”). We primarily target our recommendations at sponsors and investigators, although regulators, IRBs, PWLC and others might find them helpful in considering these trials.

## The ethical imperative to conduct more trials of disease-modifying treatments

Others have advocated for more treatment trials on Long COVID, variously highlighting their scientific and potential clinical benefits and the potential harms of PWLC seeking experimental therapies outside of trials.^6^^,^^31^, ^32^, ^33^, ^34^, ^35^, ^36^, ^37^, ^38^, ^39^, ^40^ We build on these points to provide the first comprehensive ethical argument for why more trials of disease-modifying treatments are needed now.

First, the high disease burden of Long COVID, lack of disease-modifying treatments (Table 1) and paucity of trials of such treatments (Appendix 1,2) creates significant urgency to conduct more treatment trials.^6^^,^^31^^,^^33^, ^34^, ^35^, ^36^, ^37^, ^38^, ^39^, ^40^

Second, based on many observational studies on Long COVID,^23^^,^^33^^,^^34^^,^^39^ our understanding of the natural history of Long COVID, disease pathophysiology, and clinical phenotypes, is rapidly expanding.^6^^,^^38^ While the pathophysiology of Long COVID remains incompletely understood,^4^^,^^33^^,^^35^ the knowledge gained has helped identify possible disease-modifying treatment targets, including several types of immunomodulatory, neurological, antiviral and other drugs, as well as some other therapeutic approaches such as devices.^50^

Third, some of the identified disease-modifying treatment candidates would likely have a reasonable risk-benefit profile for participants in clinical trials, especially in trials involving PWLC with high unmet disease burdens. For example, some treatment candidates are widely used to treat diseases other than Long COVID and are expected to be equally safe in PWLC, even if the evidence on the given agent’s potential clinical benefits for PWLC is still emerging (e.g., monoclonal antibodies, several immune modulators, GLP-1 agonists). Other novel treatments that the available evidence suggests have limited risks, or a higher chance of being effective, may also have a reasonable risk-benefit profile for participants.

Fourth, testing more disease-modifying treatments can have major scientific and social value at this time. Positive results from small, proof-of-concept trials can provide the rationale to conduct larger trials and attract much needed private investment in Long COVID. Positive results from large, randomized trials might support regulatory licensure. Negative results can reveal unforeseen safety issues. In some cases, even negative studies can help identify subgroups of patients in whom there may be a treatment response since Long COVID may have multiple pathophysiological subtypes. Thus, both positive and negative trial results can yield insights about Long COVID or the given therapeutic agent that advance future research.^33^^,^^38^^,^^39^ For example, if intravenous immunoglobulin succeeded in clinical trials, this could point to an immunologic cause of Long COVID,^51^ and could direct research studies into cheaper, better, and more effective treatments, much as happened in other neuroinflammatory conditions and/or autoimmune conditions.^52^ Conversely, although a small trial of SARS-CoV-2 monoclonal antibodies did not show improvement in Long COVID symptoms, a secondary pharmacokinetic analysis revealed benefit in a subset of participants with low baseline antibody levels and high drug exposure, supporting a more aggressive dosing strategy in future trials.^53^ And the failure of antiviral agents in Long COVID, notably Paxlovid,^54^^,^^55^ suggests that replication competent virus may not be present in tissue reservoirs and thus may not be the driver of the illness. Therefore, while the anticipated social value of each individual treatment trial might be lower now than in the future when our understanding of Long COVID is more advanced, the aggregate social value of conducting more trials of disease-modifying treatments now would be significant.

Fifth, given the above, it is ethical to increase investment in these trials. Of course, limited research budgets for Long COVID might mean that funding more treatment trials displaces funding for other valuable studies. Some argue for increasing funding for Long COVID research.^32^^,^^39^^,^^56^^,^^57^ However, because research budgets are limited, it is ethically imperative to optimize the scientific and social value of funded research.^47^ In our view, a pivot to more, well-designed, trials of disease-modifying treatments for Long COVID follows from this imperative.

Additionally, without investing in more treatment trials, clinicians and PWLC may, out of desperation, continue to use unproven medicines outside of trials.^20^^,^^37^^,^^58^^,^^59^ Offering experimental treatments outside of research to benefit individual PWLC can be appropriate under certain conditions (e.g., when PWLC are not eligible for clinical trials), if done under close medical supervision.^60^^,^^61^ However, using experimental treatments outside of research exposes PWLC to risks with limited oversight. Moreover, any collected data have serious limitations in terms of its validity and generalizability. PWLC need and want opportunities to try promising treatments in trials that protect their rights and interests, while also moving the field forward.

Taken together, there is a strong ethical case that the time is ripe for more trials of disease-modifying treatments for all PWLC, including groups who might require special protections in clinical trials. For example, in our view, more treatment trials in children are needed now because children experience a high disease burden from Long COVID and some of the identified disease-modifying treatments are likely to have a reasonable risk-benefit profile for them in clinical trials (e.g., low-dose naltrexone).^62^, ^63^, ^64^ Moreover, delaying trials in children may risk wide implementation of treatments based on adult data only, whereas pediatric trials are needed to rigorously assess candidate treatments in children given that clinical outcomes in children may differ from adults.

## Ethical challenges and proposed solutions in treatment trials

Conducting more treatment trials at this time poses foreseeable ethical challenges. Specifically, the urgency to develop disease-modifying treatments and current scientific uncertainties around Long COVID can make implementing trials consistent with recognized ethical considerations for clinical research challenging.^65^^,^^66^ These challenges are likely to persist even if—as we assume throughout this section—sponsors, investigators and others scrupulously review the available evidence from preclinical, observational and pilot studies^67^^,^^68^ and ongoing trials and use this evidence to support their decision-making.

Furthermore, treatment trials can be complicated by substantial disagreement about research priorities^31^^,^^33^^,^^39^^,^^40^^,^^49^^,^^56^^,^^69^, ^70^, ^71^, ^72^, ^73^, ^74^ and trial design^33^^,^^40^^,^^49^^,^^71^ among sponsors, investigators, PWLC and others interested in treatment development for Long COVID. Some PWLC also harbor mistrust of the medical establishment. This is understandable because, although recognition of Long COVID as a clinical entity among the medical establishment has been improving, many PWLC have had or continue to have negative interactions with healthcare providers (e.g., ‘medical gaslighting’^13^, ^14^, ^15^, ^16^^,^^75^, ^76^, ^77^, ^78^, ^79^). While collaborative partnership with relevant parties is generally important in clinical research,^66^ it is critical for successfully pivoting from foundational research on Long COVID to more trials of disease-modifying treatments. Specifically, engaging PWLC^6^^,^^13^^,^^32^^,^^36^^,^^44^^,^^45^^,^^73^^,^^77^^,^^78^^,^^80^, ^81^, ^82^ and experts across multiple organ systems affected by Long COVID^4^^,^^9^^,^^13^^,^^20^^,^^39^^,^^68^^,^^77^ and related illnesses,^32^^,^^68^^,^^81^ regulators, and relevant actors in health systems (e.g., policymakers, payers) can improve the quality of decision-making about research priorities and trial design,^6^^,^^13^^,^^73^^,^^77^^,^^78^^,^^80^^,^^81^ improve the coordination of research efforts,^39^ and facilitate future clinical implementation.^31^^,^^33^^,^^68^^,^^83^^,^^84^ Engaging with PWLC is also valuable in its own right and can help build trust.^45^^,^^85^ Throughout this section, we therefore assume that sponsors, investigators and others engage relevant parties, use best practices for meaningful engagement especially of PWLC,^46^^,^^86^ leverage best practices for managing disagreement,^85^^,^^87^^,^^88^ and are as transparent as possible.^31^^,^^71^^,^^85^

Even so, some disagreements around how to move Long COVID research forward will likely persist because they involve ethical trade-offs that individuals and groups may make differently. Combined with the need to prioritize limited research funding and significant public and political scrutiny of Long COVID, this may put pressure on sponsors, investigators and others (e.g., to design studies to be maximally inclusive or test specific interventions). This pressure may in some instances be conducive, and in others be a barrier, to optimizing the prioritization, design, conduct, and dissemination of treatment trials. In this setting, it is especially important to justify the process and content of decisions that involve ethical trade-offs. This section aims to equip sponsors, investigators and others in this regard.

In what follows, we discuss the most pressing ethical challenges in conducting more Long COVID treatment trials. We make concrete suggestions for how sponsors, investigators and others can address these challenges, to show that conducting more treatment trials is ethically feasible at this time. We focus on four ethical considerations, but assume that treatment trials adhere to the other recognized ethical considerations, such as independent review.^65^^,^^66^ Using evidence-based approaches to obtaining participants’ informed consent (e.g., extended person-to-person discussion, test-feedback approaches during the informed consent process^89^^,^^90^) is particularly important in light of the uncertainty and disagreements about trial design.

### Social value

Clinical trials should have sufficient social value to justify exposing participants to risks and burdens and using limited resources.^65^^,^^66^ Specifically, trials should generate data that can be used to improve human health or advance scientific knowledge.^91^ Social value is also a key criterion for deciding which treatment candidates to prioritize for trials when research budgets are limited.^47^

As argued above, conducting more treatment trials can have major social value at this time in aggregate. However, current scientific uncertainties and disagreement can make it challenging to select the best treatment candidates and achieve high social value for any particular trial. The incomplete understanding of Long COVID’s pathophysiology limits the likelihood that any particular candidate will turn into a marketed product and makes it challenging to prioritize the most promising treatment candidates for clinical testing.^20^ This challenge is further increased by the possibility that Long COVID represents several, potentially overlapping, endotypes with distinct pathophysiological mechanisms which require distinct disease-modifying treatments, as any given treatment candidate may benefit an unknown proportion of PWLC.^20^^,^^32^^,^^38^^,^^68^^,^^84^ For example, PWLC with post-Covid chronic kidney disease likely need different treatments than PWLC with anosmia and fatigue. While unknown endotypes exist in other fields (e.g., oncology), the heterogeneous pathophysiology in Long COVID^38^ creates complex challenges which distinguishes it from most other diseases. Regulatory uncertainty may additionally impact the ability to realize the anticipated social value. For example, it could be challenging to meet the FDA’s approval criteria that “the method of selection of subjects provides adequate assurance that they have the disease or condition being studied,” or “the methods of assessment of subjects’ response are well-defined and reliable” (21CFR 314.126). Finally, as mentioned, there are disagreements about which candidates to prioritize for clinical trials when funding is insufficient to test all valuable investigational agents.^31^^,^^33^^,^^39^^,^^40^^,^^49^^,^^56^^,^^69^, ^70^, ^71^, ^72^, ^73^, ^74^

We propose that funders, sponsors, investigators and others consider addressing these challenges related to selecting treatment candidates for clinical testing and optimizing the social value of individual trials as follows.

When selecting treatment candidates for clinical trials, sponsors, investigators and others should only consider treatment candidates with a reasonable level of supportive evidence (see also section on favorable risk-benefit ratio). Moreover, their choices should be informed by recent ethical guidance on research priority-setting.^47^^,^^85^^,^^92^ Three questions are especially salient for evaluating social value and choosing which treatments to study first.

First, if trials of a given treatment candidate have positive results, how likely are these results to be translated into clinical benefits for PWLC, and what is the magnitude of the anticipated benefits? Among other considerations, the answer to this question will depend on the expected risk-benefit profile of the treatment candidate in light of the available evidence. It will also depend on the ease of widely implementing the given candidate, if found to be safe and effective, including needed infrastructure and expertise for manufacturing, product storage and treatment administration, as well as anticipated cost-effectiveness.^67^

Second, if trials of a given treatment candidate have positive, negative or mixed results, how likely are these results to inform future research that will ultimately benefit PWLC? Among other considerations, this can depend on whether the given treatment candidate can validate or refute a putative disease mechanism for Long COVID, and what additional data can be collected (e.g., to help identify biomarkers or clarify pathophysiology). As already mentioned, negative trials results can provide important insights into the pathophysiology of Long COVID and inform future research.

Third, which subpopulations stand to benefit from the proposed clinical trial? Sponsors, investigators and others may have to choose among Long COVID trials that benefit different PWLC subgroups (see also section on fair participant selection). Different decisions may be justifiable, for example prioritizing treatment candidates that are likely to benefit PWLC subgroups who are worse off,^93^ or those likely to provide the largest amount of benefit across groups,^94^ or those likely to benefit groups for which decision-makers have special obligations^95^ (e.g., a nonprofit with a mission to help children may prioritize trials that help children).

Comparing the social value of different treatment candidates along these lines can illuminate potentially contested cases. For example, according to some, there is limited social value in testing treatment candidates that are already widely used by PWLC.^49^^,^^96^ Yet if this use is not evidence-based, confirming or debunking the safety or effectiveness of widely used treatments can have important social value. Similarly, although a cure for Long COVID is the ultimate goal,^36^^,^^40^ when considering the likelihood and magnitude of anticipated benefits, some potential cures may be less promising than non-curative disease modifying therapies. And while retesting treatment candidates has limited social value if the goal is to find small improvements in symptoms that prior trials were not designed to detect,^36^ there can be important social value in retesting candidates if prior trials turned out not to be adequately designed (e.g., insufficient dose or improper participant selection).

Overall, funders, sponsors, investigators and others should compare the potential clinical and scientific benefits of testing different treatment candidates and select those candidates that optimize the aggregate social value of Long COVID treatment trials. This can include efforts to balance the overall trial portfolio.^44^ For example, funders might aim for a mix of treatment candidates that have significant potential clinical benefits and those that could yield transformative scientific insights. They may also opt for a mix of adult and pediatric trials, as the latter may be needed to target age-specific symptom clusters.

Besides carefully selecting treatment candidates for clinical testing, sponsors, investigators and others should design and implement individual trials in ways that optimize their social value without unduly compromising other ethical considerations for clinical research.^65^^,^^66^ Box 1 describes strategies for optimizing the social value of treatment trials during trial design.

Box 1 **Possible strategies for optimizing the social value of Long COVID treatment trials during trial design and implementation (including expediting their conduct).**

• Leverage knowledge from research on related infection-associated chronic conditions in designing treatment trials, while carefully evaluating to what extent knowledge on related conditions applies to Long COVID.^31^^,^^68^^,^^81^^,^^97^
  ○ Conversely, consider including comparator groups of persons with related conditions in treatment trials to advance knowledge on these conditions,^36^^,^^49^^,^^97^ provided this does not undermine the scientific validity of the results on Long COVID and is feasible (e.g., feasible sample size)
• Consider trial designs and data analysis methods that can maximize efficiency and possibly flexibility (e.g., adapt trial design based on emerging data).^83^
  ○ Platform trials (i.e., randomized-controlled trials in which several treatments are assessed in multiple arms against a shared control arm) can have advantages with respect to standardization (e.g., ability to compare treatments), efficiency of creating trial infrastructure on a larger scale (e.g., stable network of trial sites with trained teams) and the efficiency of a shared control arm (e.g., decreasing the number of participants to be enrolled and costs).^20^^,^^73^^,^^98^
  ○ Adaptive designs may enhance efficiency, reduce the number of participants that are randomized to a less promising treatment or dose and facilitate gaining deeper insights^99^; however, concerns about rigor (e.g., introducing bias) should be carefully addressed.^100^
  ○ Interim data analysis may protect future trial participants from receiving unsafe and/or ineffective treatment candidates and avoid waste of resources; however, they may also prevent the identification of treatment candidates that may be effective in certain subgroups (see section on “favorable risk-benefit profile”).
• Design trials to collect useful observational data (e.g., natural history or pathophysiology of Long COVID, potential biomarkers, deep characterization of participants’ clinical status),^36^^,^^49^ to increase knowledge about Long COVID with potential additional relevance to other post-viral illnesses (e.g., myalgic encephalomyelitis/chronic fatigue syndrome).^33^^,^^38^^,^^74^ However, the added value of this information should be carefully weighed against any additional burden and risks to participants.
• Define eligibility criteria and design recruitment strategies in ways that enhance generalizability (e.g., include participants from potentially clinically distinct PWLC subgroups, demographically diverse participants,^101^ and other groups with potentially distinct risk-benefit profiles such as pregnant persons, immunocompromised persons or children and adolescents^102^),^44^ provided this does not undermine the trial’s scientific validity, risk-benefit profile, and protections for participants are appropriate.^102^
• Engage PWLC on trial design (e.g., to ensure outcome parameters are meaningful to them^71^).^6^^,^^13^^,^^73^^,^^77^^,^^78^^,^^80^^,^^81^
• Build scientific collaborations (e.g., between researchers, regulators, pharmaceutical industry) to coordinate and accelerate research efforts and smooth treatment implementation.^31^^,^^33^^,^^68^^,^^83^^,^^84^ For example:
  ○ Engagement with FDA may increase the likelihood of treatment approval, while also optimizing approval times and allowing researchers to take advantage of FDA’s expertise in trial design or outcome measures meaningful to PWLC, among others.
  ○ Collaboration among researchers (e.g., trial network) may accelerate the uptake of emerging scientific insights and allow researchers to constructively scrutinize each other’s work^39^; funders or research institutions may consider supporting trial networks and/or providing incentives to collaborate.^103^
  ○ As Long COVID does not fall solely within one medical specialty, consider involving different types of clinical expertise to more comprehensively address the syndrome.^4^^,^^9^^,^^13^^,^^20^^,^^39^^,^^68^^,^^77^
• Rapidly share trial findings (e.g., through preprints) to reduce uncertainty, while maintaining high scientific standards.
• Report how Long COVID was defined^39^ and use consensus definitions where possible, to reduce current challenges caused by the use of heterogeneous definitions in meaningfully comparing trial data.^39^^,^^44^
• Analyze differences in the effectiveness of interventions across demographics or relevant groups (e.g., clinically distinct phenotypes, onset or duration of symptoms, severity of impairment, presence of co-morbidities).^44^^,^^68^
• Responsibly share data^31^^,^^39^^,^^83^^,^^104^ and optimize the reuse of shared data (e.g., by adhering to the FAIR principles^105^ and the development common data elements^44^^,^^49^).

### Scientific validity

Scientific validity is an important ethical consideration because research results can be used to improve human health or advance scientific knowledge only if they are scientifically valid, reliable and interpretable.^65^^,^^66^^,^^104^ Upholding scientific validity may also help to improve trust in Long COVID research among PWLC and the public.^101^

Several scientific uncertainties around Long COVID can make it challenging to design and conduct rigorous treatment trials. Specifically, the current diagnostic limits and uncertainties about pathophysiology pose challenges for defining inclusion criteria and establishing treatment effectiveness. For example, inclusive, symptom-based definitions of Long COVID like the one proposed by NASEM may have low diagnostic specificity, as NASEM itself recognized.^12^ If such definitions underpin trial inclusion criteria, persons with conditions other than Long COVID but similar symptoms might be included, making it more challenging to detect treatment effectiveness.^38^^,^^44^ Additionally, to the extent that Long COVID may represent many, potentially overlapping, endotypes with distinct pathophysiological mechanisms,^20^^,^^32^^,^^38^^,^^68^^,^^84^ treatment effects might be masked in trials that involve participants with multiple endotypes.^20^^,^^84^ Selecting outcome measures to evaluate treatments is equally challenging, as there currently are validated tests for certain functional outcomes but no objective biomarkers for Long COVID,^20^ and what outcome measures to use is actively debated.^33^^,^^49^ There are also areas of disagreement about trial design,^33^^,^^40^^,^^49^^,^^71^ such as the use of placebo controls that some PWLC reject because they feel being enrolled in the placebo arm can prolong or add to their suffering.^49^ Finally, the urgency to develop treatments for Long COVID may lead investigators to make methodological choices that expedite timelines but “cut scientific corners”,^33^ a concern familiar from the early phase of epidemics and pandemics.^106^

We propose that sponsors and investigators address these challenges as follows. First, even in the face of scientific uncertainties, sponsors and investigators should define inclusion criteria for treatment trials based on scientific, risk-benefit, and fairness considerations (see also section on fair participant selection).^48^^,^^65^^,^^66^^,^^104^ In terms of scientific considerations, investigators should identify the scientific goals of the given trial and, to the extent that this does not jeopardize the trials’ ability to meet those goals, open trials to all PWLC groups that could benefit from the generated knowledge. For example, if existing evidence suggests that a given treatment candidate will only benefit PWLC with a specific putative endotype of Long COVID, the trial should test the candidate’s safety and efficacy in PWLC with this endotype. Broader inclusion criteria could jeopardize the scientific validity of the results, while also exposing PWLC with other endotypes to the risks of a treatment candidate that is not expected to be beneficial for them. Conversely, if a given treatment candidate is less clearly linked to a specific endotype of Long COVID, scientific and fairness considerations support testing this candidate in a broader group of PWLC. In this case, broader inclusion criteria would allow investigators to learn more about the given treatment candidate and the pathophysiology of Long COVID, while PWLC who could reasonably benefit would be eligible to enroll.^20^^,^^84^ For instance, the trial could enroll PWLC with the major endotypes and analyze treatments effects in each endotype. We appreciate that some PWLC advocate for maximum inclusivity in treatment trials (e.g., considering past and current stigmatization and the need to develop treatments for all PWLC),^49^^,^^97^ and we generally endorse opening trials to all PWLC who could reasonably benefit.^48^ However, limiting inclusion can be necessary in some trials (e.g., those that target specific pathways or molecules) in order to obtain scientifically valid results, which fundamentally justify the risks and costs of research.

Second, sponsors and investigators should consider innovative trial designs that are scientifically rigorous, but limit the size of the placebo group, expedite treatment development or have other advantages (Box 1). Except in small trials that explore early signals of clinical benefit, randomization to a concurrent control group (e.g., standard of care with placebo or no treatment) is needed to interpret trial results given the waxing and waning symptoms of Long COVID or clinical improvements without therapeutic intervention.^38^ However, platform trials with a shared control group can reduce the number of participants receiving placebo or no treatment and save time overall.^20^^,^^73^^,^^98^ Similarly, cross-over designs allow all participants to receive the treatment candidate being studied, while serving as their own controls as well as the control group for others.^36^^,^^38^ To the extent that innovative designs raise concerns about scientific validity (e.g., adaptive designs might introduce bias), these should be carefully addressed.^100^

Third, as validated outcome measures for Long COVID currently do not exist,^20^ sponsors and investigators should select outcome measures based on scientific evidence and PWLC values; a mix of measures to assess biology, symptoms and quality of life is generally preferable at this time.^40^^,^^104^ Investigators may use relevant measures used for diseases other than Long COVID, although these might need to be adapted and validated in PWLC.^35^ In pediatric trials, using age-specific validated outcome measures is important,^19^ as well as monitoring change in symptoms as children develop and transition across age groups. When possible, trials would ideally embed pharmacokinetic and mechanistic studies that allow for validating their outcome measures.

Fourth, given current scientific uncertainties around Long COVID, sponsors and investigators should design trials in ways that advance scientific knowledge regardless of whether the main outcomes for a given treatment candidate are positive. For example, investigators might collect data on potentially relevant characteristics of the study population that could explain treatment outcomes or allow the analysis of outcomes in different groups (e.g., currently known endotypes of Long COVID, onset or duration of symptoms, severity of impairment, presence of co-morbidities^68^ including ones that may be underdiagnosed, potential social determinants of Long COVID,^24^^,^^25^^,^^44^ and other clinical characteristics by which PWLC are suspected to differ^4^). Similarly, investigators might collect and analyze data on possible confounders of treatment outcomes, such as SARS-CoV-2 re-infection^36^ or any use of unproven or complementary medicines or supplements that could interact with investigational treatments.^107^ In pediatric trials, analyzing age-based subgroups can be important as Long COVID symptoms might differ by age. Even if trials have negative results, they can help answer important questions (e.g., effect of re-infection on disease burden and pathophysiology). While optimally designing trials to advance scientific knowledge about Long COVID is important, investigators should minimize the burden and risks to participants from any additional research procedures and ensure that the social value of the knowledge they are seeking justifies the risks (see section on favorable risk-benefit ratio).

### Fair participant selection

Fair participant selection requires that scientific and fairness considerations determine who is recruited and enrolled in clinical research.^65^^,^^66^^,^^104^ To the extent the scientific goals of clinical trials allow, investigators should open trials to all PWLC groups that could benefit from the generated knowledge (so this knowledge ‘fairly’ benefits all relevant groups); give potential participants a fair opportunity to participate in trials that offer potential clinical benefits; and ensure that the risks and burdens of research participation are shared fairly.^44^^,^^48^

All aspects of fair participant selection pose challenges in Long COVID treatment trials. Given current scientific uncertainties around Long COVID,^4^^,^^33^^,^^35^ it can be difficult to determine which groups could benefit from the knowledge gained from treatment trials.^44^ Moreover, enrolling representative samples of PWLC who could reasonably benefit, including the most affected populations (e.g., PWLC who may be severely disabled and homebound), can require time and limited resources and potentially delay trial results or preclude the conduct of additional trials—a tension that can go unrecognized in the Long COVID literature. It can also be challenging to determine when PWLC have a fair opportunity to participate, for example when they require substantial accommodation for serious symptoms or when trials are in high demand,^49^ meaning there are more eligible and willing participants than available slots because of the currently high number of PWLC with limited therapeutic options and research opportunities. Finally, there is disagreement about how to address some of these challenges.^49^^,^^97^

To address these challenges, sponsors and investigators should consider the following. First, we generally endorse opening treatment trials to all PWLC who could reasonably benefit^44^^,^^48^ especially those groups that tend to be excluded. For example, sponsors and investigators should systematically evaluate the inclusion of children and adolescents in trials,^19^^,^^104^^,^^108^ especially when the symptoms targeted by the given treatment candidate are similar across the life course.

However, as discussed above, inclusion criteria might need to be limited to obtain scientifically valid trial results (see section on scientific validity). Similarly, risk-benefit considerations can justify or further support limits on inclusion criteria for treatment trials (see section on favorable risk-benefit profile). For example, excluding PWLC who have specific comorbidities or use certain medicines or supplements can be appropriate^107^ if the available evidence suggests that the investigational treatment would pose excessive risks to participants or jeopardize the scientific validity of the results. Additionally, sponsors and investigators might justifiably limit inclusion criteria if it would take too much time or resources to enroll enough participants in the relevant subgroups to evaluate differences in treatment outcomes, and combining the subgroups may not allow evaluating the primary outcomes. For example, although scientific and fairness considerations might support enrolling PWLC with all of the major endotypes in some treatment trials, as we suggested above, the required sample sizes might be too large to justify such broad inclusion. In a similar vein, although people with infection-associated chronic conditions other than Long COVID might reasonably benefit from being included in some trials (e.g., as an additional comparator group where scientifically appropriate^36^^,^^49^^,^^97^), sponsors and investigators might limit inclusion to PWLC to exercise stewardship of limited resources. As long as resources are limited and developing disease-modifying treatments for Long COVID remains urgent, sponsors and investigators should carefully consider whether the social value of using broader inclusion criteria justifies any additional time and resources needed to complete a given trial. How to balance these trade-offs may depend on the trial and context; for example, ensuring representativeness may be more important in large RCTs than in small proof-of-concept trials.

Second, sponsors and investigators should reduce barriers to accessing treatment trials^4^^,^^6^^,^^44^ to ensure that eligible PWLC have a fair opportunity to participate. This can involve widely publicizing trials, engaging PWLC to identify and address barriers to participation (e.g., home or telehealth visits for home-bound PWLC, if sufficiently safe and rigorous),^44^ selecting trial sites to enhance access to trials^44^ (e.g., including the relative merits of international sites^109^) and improving trial infrastructure (e.g., establishing referral relationships^110^).^83^ Beyond reimbursement for research-related expenses, sponsors and investigators might also consider paying participants to facilitate recruitment; while sometimes controversial, payment for research participation can be ethically appropriate and even desirable.^44^^,^^111^ However, just like using broader inclusion criteria, giving all eligible PWLC a fair opportunity to participate in treatment trials can require time and resources and potentially compromise the scientific rigor of collected data. For instance, PWLC can be sensitive to stressors that most people experience as trivial and require special accommodations, such as sensitivity to the orthostatic stress of sitting in a chair, requiring space to lie down in waiting and consultation rooms. As another example, offering telehealth or home visits may enhance accessibility (e.g., home–or bedbound PWLC), but involve trade-offs relating to data quality (e.g., because of limitations of available equipment, freshness of samples). Again, we do not believe there is a single approach to resolving possible tensions between promoting fair opportunity, exercising stewardship of limited resources, ensuring scientific validity and obtaining timely trial results.

Third, sponsors and investigators should anticipate that some trials might be in high demand^49^ and develop a transparent process for how to select participants fairly, so as to minimize the potential biases of ad-hoc decision-making (e.g., bias favoring well-connected PWLC).^46^ Experience from other trials shows that selection among eligible participants can promote different ethical considerations, such as enhancing the trial’s social value, optimizing the risk-benefit profile for participants or promoting justice or fairness—but generally not all of these considerations simultaneously.^46^ Depending on the trial, some approaches are more justifiable than others.^46^ It is also possible that otherwise controversial inclusion criteria might become more acceptable when trials are in high demand. For example, it remains contested, including among these authors, whether laboratory-confirmed SARS-CoV-2 infection is an acceptable requirement for inclusion.^6^ A confirmed diagnostic test likely reduces the chance of enrolling people without Long COVID but with other conditions, for whom the trial involves risks but no prospect of benefit and whose enrollment might negatively impact the trial’s scientific validity. However, laboratory tests were difficult to access early in the pandemic, especially for certain populations, and rapid self-tests later became a reasonable alternative.^44^ These concerns might be less significant when trials are in high demand and, for example, a weighted lottery gives PWLC with confirmed infection a higher chance of being enrolled to enhance the trial’s scientific validity.

### Favorable risk-benefit ratio

Risks to participants should be minimized and outweighed by the potential clinical benefits for participants and the social value of the given trial (making the overall risk-benefit profile ‘favorable’).^65^^,^^66^ Given the high unmet disease burden of Long COVID, the potential clinical benefits of investigational treatments can be significant.

However, ensuring that treatment trials have a favorable risk-benefit profile can be challenging for several reasons. As previously discussed (see sections on social value and scientific validity), our currently incomplete understanding of Long COVID, diagnostic limitations, and the possibility of several endotypes creates significant uncertainty about the likelihood that the potential benefits of a treatment candidate will outweigh the risks in known or unknown groups of PWLC. Given the symptoms of Long COVID, the risks and burdens of trial participation may also be higher for PWLC than for other populations. For example, hospital visits might exacerbate symptoms.^36^ While discussions about Long COVID emphasize the importance of minimizing the burdens of trial participation,^36^^,^^49^ doing so can come into tension with other ethical considerations, such as collecting additional data to optimize the social value of a given trial.

As in all clinical treatment research, sponsors and investigators should prioritize treatment candidates with a favorable risk-benefit profile and minimize risks as much as possible. Trials of candidates with very uncertain clinical benefits may only be acceptable if the available evidence suggests limited risks (e.g., repurposed treatments with an good safety record in other populations that is expected to be equally safe in PWLC, novel treatment candidates that raise limited safety concerns). Higher risks may be acceptable, within limits, when treatment candidates have substantial potential clinical benefits in PWLC who suffer from severe symptoms despite receiving the best available supportive care. In pediatric trials with a prospect of benefit, U.S. regulations generally require that risks are justified by the anticipated benefits to participants, and that the risk-benefit profile of the treatment be at least as favorable as existing alternatives (45 CFR 46.405a/b). This will often favor treatments that are either already used in pediatric clinical practice or have significant supportive data from adults. However, testing novel treatment candidates in children and adolescents can be justifiable and avoid delays. Furthermore, as described in fair participant selection, groups facing higher risks may in some cases justifiably be excluded (e.g., with certain comorbidities).

Second, after selecting the treatment candidate and study population, sponsors and investigators should consider various strategies for optimizing the risk-benefit ratio of trials for participants that carefully balance different ethical considerations. For example, offering telehealth or home visits can reduce burdens on home–or bedbound PWLC, while also giving them a fair opportunity to participate.^36^^,^^49^ However, such visits may only be acceptable if they do not introduce their own safety risks for participants (e.g., treatment candidate with a significant risk of severe anaphylactic reactions that can only be addressed in medical settings) and do not unduly compromise the social value or scientific validity of the trials.^112^ Please see Table 2 for additional strategies for optimizing the risk-benefit profile of treatment trials for participants.

**Table 2:** Possible strategies for optimizing the risk-benefit ratio of Long COVID treatment trials for participants.

Minimizing risks and burdens	• Explore opportunities to minimize risks of investigational treatments (e.g., monitor participants who use other medications or supplements for potential drug–drug interactions; discontinuation of such medications or supplements may be necessary to reduce risks to participants, although any negative impacts on them should be carefully considered) and trial participation (e.g., take appropriate measures to reduce risk of re-infection during study visits). • Balance the need to minimize the burdens for participants (which also enhances accessibility) with potential impacts of such design choices on the trial’s risk-benefit profile and social value; efforts to minimize burdens should not jeopardize the scientific goals of the trial or significantly increase risks. Communicate clearly to potential participants about what can be accommodated. ○ For example, offering telehealth or home visits may reduce burdens, enhance accessibility (e.g., home–or bedbound PWLC), and has the advantage of observing the person in their home environment which provides a better assessment of their functional abilities and is hence strongly argued for by some.^36^^,^^49^ However, in person visits can be necessary for physical and neurological assessments which may also require sophisticated equipment and for obtaining biological samples particularly those that need to be processed over a short interval. This is particularly important in PWLC since many of the immune and virological assays require fresh samples. Televisits may also negatively impact safety and the social value or scientific validity of some trials^112^ (e.g., therapies with a relatively high risk of severe anaphylactic reactions should only be given in settings with medical resources).
Enhance potential benefits	• To enhance potential clinical benefits for participants, consider trial designs that eventually give more, or all, participants access to promising treatments under study.^36^^,^^38^ ○ For example, if this does not interfere with scientific validity, consider placebo-controlled cross-over designs or designs that randomize a smaller proportion of participants to the control group (e.g., a shared control arm in platform trials). ○ Alternatively, plan to facilitate posttrial access for participants who benefit and control groups if treatments are proven beneficial. • Explore ways to maximize ancillary benefits of trial participation for participants (e.g., diagnosis of potentially treatable comorbid conditions, monitoring by a clinician, reporting of incidental findings if clinically relevant). ○ Current difficulties in accessing clinical care^14^^,^^15^^,^^77^^,^^78^^,^^113^ may make ancillary benefits especially valuable to PWLC, which underscores the importance of announcing the opportunity to participate widely (e.g., traditionally difficult to reach groups).
Characterizing risks and burdens	• Consider the risks and potential benefits of trial participation for children and adolescents separately from those for adults, as Long COVID has both similar and distinct symptom clusters in children and adolescents^10^ and investigational treatments may therefore have different clinical effects. ○ For example, risk-benefit profiles might be more favorable for adults than for children because of heightened uncertainty around Long COVID in children (e.g., more limited understanding of physiology, clinical presentation and pathophysiology) and the paucity of data on treatments that are used for other conditions in children and may be repurposed for Long COVID. In contrast, some interventions may have a more favorable risk-benefit profile for children than for adults, as they may target age-specific symptom clusters. • When designing trials, engage PWLC to help characterize the risks, potential benefits, and burdens,^44^ as well as the trials’ acceptability as PWLC have unique insights that can enhance the quality of the trial. ○ For example, engaging PWLC may help characterize the distinct risks and burdens of trial components for PWLC (e.g., the risks of cardiopulmonary exercise for PWLC with post-exertional malaise) • Set up a monitoring plan for risks, burdens, and potential benefits of trials (e.g., post exertional malaise^49^^,^^97^) and adapt if necessary. ○ Independent scientific advisors or data and safety monitoring boards (DSMBs) with the appropriate expertise (e.g., pediatric expertise for trials with children) could perform the monitoring.

## Future directions

We hope that the suggestions offered here will help investigators and reviewers promote the ethics of these important trials, helping advance the interests of research participants while bringing much-needed disease-modifying therapies to the Long COVID community with rigor and expediency. Our analysis and recommendations will need to be revisited as Long COVID treatment trials progress, since changing circumstances may change ethical trade-offs or raise new ethical challenges (e.g., less scientific uncertainty with increasing knowledge about Long COVID).

Finally, we believe our discussion can inform decisions about when and how to pivot from foundational observational research to clinical trials for diseases with similar challenges as Long COVID related to urgency, uncertainty and disagreement.

## Contributors

SH, CG, AN, and AR conceptualized the paper. SH, CG, and AR wrote the original draft. The draft was circulated to all authors for review. MLF, RSG, CM, MJP, SV, and AN reviewed and revised the draft critically for important intellectual content. The paper was finalized using an iterative process, until all authors’ comments had been addressed. All authors approve of the final version and agree to be accountable for all aspects of the work.

## Use of AI and AI-assisted technologies

During the preparation of this work the authors used ChatGPT/OpenAI to improve readability and language. After using this tool/service, the authors reviewed and edited the content as needed and take full responsibility for the content of the publication.

## Declaration of interests

MLF received grants or contracts from Brain Inflammation Collaborative, INC and Patient Led Research Collaborative as well as consulting fees from RECOVER (NIH). RG reports that institutional grant funding as MPI for the NIH-funded Researching COVID to Enhance Recovery (RECOVER) Initiative supported the present manuscript. CM has received consulting fees from RECOVER (NIH) and support for attending meetings and/or travel from RECOVER (FNIH) and RECOVER (NYU). MJP has received grants or contracts from NIH and PolyBio Research Foundation; consulting fees from Gilead Sciences, AstraZeneca, BioVie, Apellis Pharmaceuticals, and BioNTech; support for attending meetings and/or travel from Invivyd; and receipt of equipment, material, drugs, medical writing, gifts or other services from Aerium Therapeutics and Shionogi. All other authors declare no competing conflicts of interest.

## Appendix A Supplementary data

Appendices 1–4

## Footnotes

i Estimate based on census data that were collected Apr 30–May 27, 2024 (data label ‘April 30, 2024')99. Statistics. NCfH. Household Pulse Survey, 2022–2024. Long COVID. In: Bureau USC, editor.; total number calculated based on estimated percentage of adults currently experiencing long COVID (6.00%) and the population size on May 27, 2024 of 336,489,513 (https://www.census.gov/popclock/).
