# Supplementary material for: The next phase in Long COVID research: addressing the ethical challenges in trials of disease-modifying treatments
Source: eClinicalMedicine. 2026 May 7;95:103918. doi: 10.1016/j.eclinm.2026.103918 (PMC13185860; doi:10.1016/j.eclinm.2026.103918)
Supplement: Appendices 1–4 [file mmc1.docx]

#### **Appendix 1. Methods of rapid clinical trials registry search for trials of disease-modifying treatments for Long COVID.** A professional librarian assisted with developing the search strings and running the search.

####

| **Search date** | September 2025 |
| --- | --- |
| **Database(s)** | ClinicalTrials.gov |
| **Search string(s)** | (long Covid OR long Covid19 OR post acute sequelae of covid19 OR post acute sequelae of sars cov2 infection) AND interventional studies |
|  | Rationale: We combined terms related to “Long COVID” in the “conditions/disease” search box to capture the below list of synonyms for Long COVID. We used the term “drug treatment” in the “intervention/treatment” box to focus the search on. Finally, we filtered for interventional studies and drug treatments. Although the use of filters has limitations, we deemed this appropriate for a rapid search aimed at generating an estimate of trials of disease-modifying treatments for Long COVID in a single clinical trials database. |
|  | List of synonyms for Long COVID captured: long COVID; long COVID19; long COVID-19 syndrome; post-acute COVID-19; post-acute COVID19 syndrome; post-acute sequelae of SARS-CoV-2 infection; post-acute sequelae of COVID-19; persistent COVID-19; long haul COVID; long-haul COVID-19; long hauler COVID; post-acute COVID syndrome; chronic COVID syndrome; long hauler COVID; post-acute sequela of COVID-19. |
| **Selection criteria** | Exclusion criterion: Not a therapeutic clinical trial for Long COVID (e.g., trials testing interventions during the acute phase, diagnostic interventions).  Inclusion criterion: Clinical trial of disease-modifying treatments (defined as treatments alter the course of the disease by targeting putative pathologic mechanisms of Long COVID and hence go beyond relieving symptoms) |
|  | Rationale: We used broad selection criteria in order to capture the full range of trials of disease-modifying treatments for Long COVID. |
| **Search results** | The initial clinicaltrials.gov search yielded n=397 results, of which n=58 were excluded because they were not therapeutic clinical trials for Long COVID.  Of the 339 therapeutic clinical trials for Long COVID, n=78 studied interventions with a potential disease modifying effect. |
| **Reviewers** | Primary: Avindra Nath, Michael J. Peluso, secondary (as needed): Saskia Hendriks. |
| **Search design and summary of results** | Design: Annette Rid, Saskia Hendriks, Avindra Nath, Michael J. Peluso, Cynthia Sheffield; summary: Saskia Hendriks, Annette Rid. |

#### **Appendix 2. Results of rapid clinical trials registry search for trials of disease-modifying treatments for Long COVID.**

**List of recruiting Long COVID trials testing potentially disease modifying interventions**

| **NCT Number** | **Study Title** | **Study URL** | **Study Status** | **Conditions** | **Interventions** |
| --- | --- | --- | --- | --- | --- |
|  |  |  |  |  |  |
| NCT07123727 | A Study to Examine Anktiva for the Treatment of COVID-19. | <https://clinicaltrials.gov/study/NCT07123727> | RECRUITING | Long COVID\|Long COVID Syndrome\|Long Covid 19 | DRUG: Anktiva |
| NCT06437223 | Study of Xiflam‚Ñ¢ Treatment in Patients Post COVID-19 Infection Suffering From What is Known as Long COVID (LC) | https://clinicaltrials.gov/study/NCT06437223 | RECRUITING | Long COVID | DRUG: Tonabersat\|DRUG: Placebo |
| NCT06928272 | Long Covid (LC)-REVITALIZE - A Long Covid Repurposed Drug Study | https://clinicaltrials.gov/study/NCT06928272 | RECRUITING | Long COVID | DRUG: Pirfenidone\|DRUG: Placebo for pirfenidone\|DRUG: Upadacitinib\|DRUG: Placebo for upadacitinib |
| NCT06511063 | Antiviral Clinical Trial for Long Covid-19 | https://clinicaltrials.gov/study/NCT06511063 | RECRUITING | Long Covid | DRUG: tenofovir disoproxil/emtricitabine\|DRUG: Selzentry\|DRUG: Placebo |
| NCT05747534 | AT1001 for the Treatment of Long COVID | https://clinicaltrials.gov/study/NCT05747534 | RECRUITING | Long COVID\|Long COVID-19\|Post Acute COVID-19 Syndrome\|Post Acute Sequelae of COVID-19 | DRUG: Larazotide Acetate\|DRUG: Placebo |
| NCT06847191 | NE3107 in Adults With Neurological Symptoms of Long COVID | <https://clinicaltrials.gov/study/NCT06847191> | RECRUITING | Long COVID | DRUG: NE3107\|DRUG: Placebo |
| NCT06960928 | Low Dose Sirolimus in People With Post-Acute Sequelae of COVID-19 (PASC) Long COVID-19 | https://clinicaltrials.gov/study/NCT06960928 | RECRUITING | Long COVID-19 | DRUG: Low-dose sirolimus\|DRUG: Placebo |
| NCT06128967 | A Multicenter, Adaptive, Randomized, doublE-blinded, Placebo-controlled Study in Participants With Long COVID-19: The REVIVE Trial | https://clinicaltrials.gov/study/NCT06128967 | RECRUITING | Long COVID-19 Syndrome\|Chronic Fatigue Syndrome | DRUG: Fluvoxamine Maleate 100 MG\|DRUG: Placebo\|DRUG: Metformin Extended Release Oral Tablet |
| NCT07021794 | SARS-CoV-2 Specific Monoclonal Antibody for Post-COVID-19 Conditions (Long COVID) | https://clinicaltrials.gov/study/NCT07021794 | RECRUITING | Post-COVID / Long-COVID | DRUG: Placebo\|BIOLOGICAL: Sipavibart |
| NCT06590324 | A Study of Apabetalone in Subjects With Long -COVID | <https://clinicaltrials.gov/study/NCT06590324> | RECRUITING | Post-Acute COVID-19 Syndrome | DRUG: Apabetalone |
| NCT06492798 | Effectiveness and Safety of Mesenchymal Stem Cell Therapy in Long COVID Patients | https://clinicaltrials.gov/study/NCT06492798 | RECRUITING | Long COVID\|Post-Acute COVID-19 Syndrome | DRUG: umbilical cord mesenchymal stem cell |
| NCT06585254 | tVNS in Long COVID-19 | https://clinicaltrials.gov/study/NCT06585254 | RECRUITING | Long COVID\|Chronic Fatigue Syndrome | DEVICE: Transcutaneous vagus nerve stimulator |
| NCT05926505 | Safety and Efficacy of Anakinra Treatment for Patients With Post Acute Covid Syndrome | https://clinicaltrials.gov/study/NCT05926505 | RECRUITING | Post-Acute COVID-19 Syndrome\|Post-Acute COVID-19\|Long COVID | DRUG: Placebo\|DRUG: Anakinra 149 MG/ML Prefilled Syringe [Kineret] |
| NCT06631287 | Randomized Double-Blind Placebo-Controlled Trial EValuating Baricitinib on PERSistent NEurologic and Cardiopulmonary Symptoms of Long COVID | https://clinicaltrials.gov/study/NCT06631287 | RECRUITING | Long COVID\|Sars-CoV-2 Infection\|Coronavirus Infections\|COVID-19 | DRUG: Baricitinib\|OTHER: Placebo |
| NCT06095297 | Long COVID Brain Fog: Cognitive Rehabilitation Trial | https://clinicaltrials.gov/study/NCT06095297 | RECRUITING | Long COVID\|Brain Fog\|Cognitive Impairment\|Cognitive Dysfunction\|Post-Acute COVID-19 Syndrome | BEHAVIORAL: Processing Speed Trainingtional RehabilitationPROCEDURE: Trans-auricular Vagus Nerve Stimulation: |
| NCT05421208 | Cardiovascular Autonomic and Immune Mechanism of Post COVID-19 Tachycardia Syndrome | <https://clinicaltrials.gov/study/NCT05421208> | RECRUITING | Post-acute COVID-19 Syndrome\|Postural Tachycardia Syndrome (POTS)\|Long COVID\|SARS CoV 2 Infection | DIAGNOSTIC_TEST: Levels of inflammatory cytokine ( IL-6) in post-COVID-19 POTS |
| NCT06171152 | Study of Liraglutide (A Weight Loss Drug) in High Risk Obese Participants With Cognitive and Memory Issues | https://clinicaltrials.gov/study/NCT06171152 | RECRUITING | Multiple Sclerosis\|Long COVID\|Long Covid19\|Obese\|Obesity\|Obesity, Morbid\|Acute Leukemia in Remission | DRUG: Liraglutide Pen Injector [Saxenda]\|OTHER: Medication Diary |
| NCT06441955 | Covid-19 Long Haul Preventative and Health Promotion Care Clinical Trial Acceleration Program. | <https://clinicaltrials.gov/study/NCT06441955> | RECRUITING | COVID-19, Long Haul | DRUG: Ritonavir-Boosted Nirmatrelvir (Paxlovid)\|DIAGNOSTIC_TEST: Physiological Evaluation\|BIOLOGICAL: Moderna COVID-19 Vaccine GENETIC: Genetic (including gene transfer, stem cell and recombinant DNA) |
| NCT06597396 | Study to Investigate the Efficacy of Abrocitinib in Adult Participants with Severe Fatigue from Post COVID Condition/Long COVID | https://clinicaltrials.gov/study/NCT06597396 | RECRUITING | Post-COVID Condition\|Fatigue Symptom | DRUG: Abrocitinib\|DRUG: Placebo |
| NCT06159283 | Intravenous Immunoglobulin Replacement Therapy for Persistent COVID-19 in Patients With B-cell Impairment | <https://clinicaltrials.gov/study/NCT06159283> | RECRUITING | COVID-19 | DRUG: Immunoglobulins |
| NCT04978571 | Percutaneous Electrical Nerve Field Stimulation (PENFS) in Patients With Post Concussion Syndrome (PCS) | <https://clinicaltrials.gov/study/NCT04978571> | RECRUITING | Post-Concussion Syndrome\|COVID Long-Haul\|COVID-19 | DEVICE: percutaneous electrical nerve-field stimulation, PENFS\|DEVICE: percutaneous electrical nerve-field stimulation, PENFS (sham device)\|DEVICE: percutaneous electrical nerve-field stimulation, PENFS (COVID active device) |
| NCT05205577 | Trial of Auricular Vagus Nerve Stimulation in Painful Covid Long | https://clinicaltrials.gov/study/NCT05205577 | RECRUITING | COVID-19 | DEVICE: Tens Eco Plus |
| NCT05710770 | Immunoadsorption in Patients With Chronic Fatigue Syndrome Including Patients With Post-COVID-19 CFS | <https://clinicaltrials.gov/study/NCT05710770> | RECRUITING | ME/CFS\|Post-COVID ME/CFS | DEVICE: Immunoadsorption |
| NCT05986422 | Methylprednisolone in Patients with Cognitive Deficits in Post-COVID-19 Syndrome (PCS) | https://clinicaltrials.gov/study/NCT05986422 | RECRUITING | Post-COVID-19 Syndrome | DRUG: Methylprednisolone |

**List of active (not or not yet recruiting) Long COVID trials testing potentially disease modifying interventions**

| **NCT Number** | **Study Title** | **Study URL** | **Study Status** | **Conditions** | **Interventions** |
| --- | --- | --- | --- | --- | --- |
| NCT06597682 | Evaluating Immunomodulatory Interventions in Post-Acute Sequelae of SARS-CoV-2 InfEction | https://clinicaltrials.gov/study/NCT06597682 | NOT_YET_RECRUITING | Post-acute Sequelae of SARS-COV-2 Infection | DRUG: Prednisone\|DRUG: Budesonide/Formoterol\|DRUG: Vitamin C combined with Coenzyme Q10 oral treatment\|DRUG: Montelukast tablets oral treatment |
| NCT05877508 | Anti-SARS-CoV-2 Monoclonal Antibodies for Long COVID (COVID-19) | <https://clinicaltrials.gov/study/NCT05877508> | ACTIVE_NOT_RECRUITING | Long COVID\|Post-Acute Sequela of COVID-19\|Post-Acute COVID-19 | DRUG: AER002\|OTHER: Placebo |
| NCT06189066 | Long COVID Ultrasound Trial | https://clinicaltrials.gov/study/NCT06189066 | ACTIVE_NOT_RECRUITING | Long Covid | DEVICE: Splenic Ultrasound |
| NCT06974084 | Investigating Measurable PRO Acuity Trial (IMPACT) is a Multi-Center Randomized, Double-Blind, Placebo-Controlled Study to Evaluate the Efficacy of Maraviroc and Atorvastatin to Improve Neurocognitive and Physical Function of Subjects With Long COVID-19/Post-Acute Sequelae of COVID-19 (PASC). | <https://clinicaltrials.gov/study/NCT06974084> | NOT_YET_RECRUITING | Long COVID | DRUG: Maraviroc (MVC)\|DRUG: Atorvastatin, 10mg, 20mg, 40mg\| |
| NCT07128082 | The Long COVID Treatment Trial | https://clinicaltrials.gov/study/NCT07128082 | NOT_YET_RECRUITING | Long COVID | DRUG: Tirzepatide\|DRUG: Placebo |
| NCT07108036 | A Study to Assess Anktiva in Patients With Long Covid-19. | https://clinicaltrials.gov/study/NCT07108036 | NOT_YET_RECRUITING | Long COVID | DRUG: N-803 (IL-15 Superagonist) |
| NCT06147050 | Effect of Metformin in Reducing Fatigue in Long COVID in Adolescents | https://clinicaltrials.gov/study/NCT06147050 | NOT_YET_RECRUITING | Long COVID | DRUG: Metformin\|OTHER: Placebo |
| NCT06161688 | Ensitrelvir for Viral Persistence and Inflammation in People Experiencing Long COVID | https://clinicaltrials.gov/study/NCT06161688 | ACTIVE_NOT_RECRUITING | Long COVID\|Post Acute Sequelae of COVID-19\|Post-Acute COVID-19 | DRUG: Ensitrelvir\|OTHER: Placebo |
| NCT06637800 | Prospective, Open-label Study of Seraph 100 in Patients With Prolonged COVID (PC) | https://clinicaltrials.gov/study/NCT06637800 | NOT_YET_RECRUITING | Long COVID | DEVICE: Seraph¬Æ 100 Microbind¬Æ Affinity Blood Filter (Seraph 100) |
| NCT06907251 | Dapagliflozin for Long COVID Syndrome | https://clinicaltrials.gov/study/NCT06907251 | NOT_YET_RECRUITING | COVID - 19\|Long COVID Syndrome | DRUG: Dapagliflozin (DAPA)\|DRUG: Placebo |
| NCT06156241 | Stem Cell Study for Long COVID-19 Neurological Symptoms | <https://clinicaltrials.gov/study/NCT06156241> | NOT_YET_RECRUITING | Post-Acute COVID-19 Syndrome | BIOLOGICAL: Stem Cell |
| NCT05556733 | FMT for Post-acute COVID-19 Syndrome | https://clinicaltrials.gov/study/NCT05556733 | ACTIVE_NOT_RECRUITING | Post-Acute COVID19 Syndrome\|COVID-19 | PROCEDURE: Faecal Microbiota Transplantation |
| NCT05597800 | Nivolumab/Ipilimumab and Chemotherapy Combination in Advanced NSCLC Patients With HIV, HBV, HCV and Long Covid Syndrome | https://clinicaltrials.gov/study/NCT05597800 | NOT_YET_RECRUITING | NSCLC Stage IV\|HIV\|HBV\|HCV\|Long COVID | DRUG: Nivolumab and Ipilimumab |
| NCT06968104 | Vagus Nerve Stimulation to the Ear to Improve Symptoms in Post-COVID-19 and ME/CFS | <https://clinicaltrials.gov/study/NCT06968104> | ACTIVE_NOT_RECRUITING | Post-COVID / Long-COVID\|ME/CFS | DEVICE: transcutaneous vagus nerve stimulation\|DEVICE: transcutaneous vagus nerve stimulation |
| NCT06305793 | RECOVER-AUTONOMIC: Platform Protocol, Appendix A (IVIG) | https://clinicaltrials.gov/study/NCT06305793 | ACTIVE_NOT_RECRUITING | Long COVID\|Long Coronavirus Disease 2019 (Covid19)\|Long Covid-19 | DRUG: IVIG (intravenous immunoglobulin)\|DRUG: IVIG Placebo\| |
| NCT04948203 | Assessing the Efficacy of Sirolimus in Patients With COVID-19 Pneumonia for Prevention of Post-COVID Fibrosis | https://clinicaltrials.gov/study/NCT04948203 | ACTIVE_NOT_RECRUITING | Pulmonary Fibrosis\|COVID-19 Pneumonia\|Long COVID | DRUG: Sirolimus |
| NCT05911906 | An Open-label, Clinical Feasibility Study of the Efficacy of Remdesivir for Long-COVID. | <https://clinicaltrials.gov/study/NCT05911906> | ACTIVE_NOT_RECRUITING | SARS-CoV-2 Infection\|COVID-19 | DRUG: Remdesivir |
| NCT07184385 | A Phase III Study of Human Umbilical Cord Blood (REGENECYTE) Infusion in Patients With Post-COVID Condition | <https://clinicaltrials.gov/study/NCT07184385> | NOT_YET_RECRUITING | Long COVID\|Post-COVID-19 Condition\|Post-COVID Syndrome\|Post-COVID Condition | BIOLOGICAL: REGENECYTE\|BIOLOGICAL: Placebo |
| NCT07089719 | Bevacizumab in Post-acute Sequelae of COVID-19 : Efficacy and Safety (Pilot Study) | <https://clinicaltrials.gov/study/NCT07089719> | NOT_YET_RECRUITING | Dyspnea Caused by 2019-nCoV | DRUG: Bevacizumab Injection |
| NCT04482595 | BIO 300 Oral Suspension in Previously Hospitalized Long COVID Patients | <https://clinicaltrials.gov/study/NCT04482595> | ACTIVE_NOT_RECRUITING | COVID-19\|Long COVID\|Pulmonary Fibrosis\|Post-acute Respiratory Complications of COVID-19 | DRUG: BIO 300 Oral Suspension\|DRUG: Placebo |
| NCT06510985 | Transcutaneous Vagus Nerve Stimulation (tVNS) for Improved Recovery After Exertion. | https://clinicaltrials.gov/study/NCT06510985 | NOT_YET_RECRUITING | Post-COVID-19 Syndrome | DEVICE: transcutaneous vagus nerve stimulation\|BEHAVIORAL: Monitoring\|DEVICE: cardiowatch bracelet |
| NCT04904536 | Statin TReatment for COVID-19 to Optimise NeuroloGical recovERy | <https://clinicaltrials.gov/study/NCT04904536> | ACTIVE_NOT_RECRUITING | Neurocognitive Impairment, Mild | DRUG: Atorvastatin\|OTHER: Standard Care |
| NCT06996314 | Effects of Auricular Vagus Nerve Stimulation Combined With Slow-paced Breathing on Individuals With Postural Orthostatic Tachycardia Syndrome. | https://clinicaltrials.gov/study/NCT06996314 | NOT_YET_RECRUITING | Postural Orthostatic Tachycardia Syndrome (POTS)\|Post-acute COVID-19 Syndromes | DEVICE: TVNS + Slow-Paced Breathing (TVNS+SDB)\|DEVICE: TVNS without Breathing Training (TVNS+NB)\|DEVICE: Sham TVNS + SDB\|DEVICE: Sham TVNS + NB |

**List of *completed* Long COVID trials testing potentially disease modifying interventions**

| **NCT Number** | **Study Title** | **Study URL** | **Study Status** | **Conditions** | **Interventions** |
| --- | --- | --- | --- | --- | --- |
| NCT06316843 | Valacyclovir Plus Celecoxib for Post-Acute Sequelae of SARS-CoV-2 | https://clinicaltrials.gov/study/NCT06316843 | COMPLETED | Long COVID\|PASC Post Acute Sequelae of COVID 19 | DRUG: Valacyclovir celecoxib dose 1\|DRUG: Valacyclovir celecoxib dose 2\|DRUG: Placebo |
| NCT05576662 | Paxlovid for Treatment of Long Covid | https://clinicaltrials.gov/study/NCT05576662 | COMPLETED | Post-acute Sequelae of SARS-CoV-2 Infection\|Long COVID | DRUG: Nirmatrelvir\|DRUG: Placebo\|DRUG: Ritonavir |
| NCT05965726 | RECOVER-VITAL: Platform Protocol, Appendix to Measure the Effects of Paxlovid on Long COVID Symptoms | https://clinicaltrials.gov/study/NCT05965726 | COMPLETED | Long COVID-19\|Long COVID | DRUG: Paxlovid 25 day dosing\|DRUG: Paxlovid 15 day dosing\|DRUG: Control |
| NCT05595369 | RECOVER-VITAL: Platform Protocol to Measure the Effects of Antiviral Therapies on Long COVID Symptoms | https://clinicaltrials.gov/study/NCT05595369 | COMPLETED | Long COVID\|Long Covid19 | DRUG: Experimental: Paxlovid 25 day dosing\|DRUG: Experimental: Paxlovid 15 day dosing\|DRUG: Placebo Comparator: Control |
| NCT06305780 | RECOVER-AUTONOMIC Platform Protocol | <https://clinicaltrials.gov/study/NCT06305780> | COMPLETED | Long COVID\|Long Covid19\|Long Covid-19 | DRUG: IVIG + Coordinated Care\|DRUG: IVIG Placebo + Coordinated Care\|DRUG: Ivabradine + Coordinated Care\|DRUG: Ivabradine Placebo + Coordinated Care\|DRUG: IVIG + Usual Care\|DRUG: IVIG Placebo + Usual Care\|DRUG: Ivabradine + Usual Care\|DRUG: Ivabradine Placebo + Usual Care |
| NCT05999435 | Study of LAU-7b for the Treatment of Long COVID in Adults | https://clinicaltrials.gov/study/NCT05999435 | COMPLETED | Long COVID | DRUG: LAU-7b for 3 cycles\|DRUG: LAU-7b for 1 cycle, then placebo\|OTHER: Placebo for 3 cycles |
| NCT05911009 | To Investigate Efficacy, Pharmacodynamics, and Safety of BC 007 in Participants With Long COVID | https://clinicaltrials.gov/study/NCT05911009 | COMPLETED | Long Covid | DRUG: BC 007 or matching placebo |
| NCT05668091 | A Decentralized, Randomized Phase 2 Efficacy and Safety Study of Nirmatrelvir/Ritonavir in Adults with Long COVID. | https://clinicaltrials.gov/study/NCT05668091 | COMPLETED | Long COVID | DRUG: Nirmatrelvir\|DRUG: Ritonavir\|DRUG: Placebo |
| NCT05152849 | Efficacy, Safety, Tolerability of AXA1125 in Fatigue After COVID-19 Infection | https://clinicaltrials.gov/study/NCT05152849 | COMPLETED | Post-Acute Sequelae of SARS-CoV-2 (PASC) Infection | DRUG: AXA1125\|DRUG: Placebo |
| NCT05954325 | Immunoadsorption vs. Sham Treatment in Post COVID-19 Patients With Chronic Fatigue Syndrome | https://clinicaltrials.gov/study/NCT05954325 | COMPLETED | Fatigue\|Post-Acute COVID-19 Syndrome | PROCEDURE: Immunoadsorption vs. sham immunoadsorption |
| NCT05592418 | Study to Evaluate the Efficacy and Safety of Ampligen in Patients With Post-COVID Conditions | https://clinicaltrials.gov/study/NCT05592418 | COMPLETED | Post COVID-19 Condition\|Long COVID | DRUG: Rintatolimod\|OTHER: Placebo / Normal Saline |
| NCT05608629 | Vagus Nerve Stimulation as Treatment for Long Covid | https://clinicaltrials.gov/study/NCT05608629 | COMPLETED | Long COVID\|Chronic Fatigue Syndrome | DEVICE: Transcutaneous Non-Invasive Vagus Nerve Stimulation |
| NCT04944121 | Phase 2 Study of RSLV-132 in Subjects With Long COVID | https://clinicaltrials.gov/study/NCT04944121 | COMPLETED | Post-acute Corona Virus 19 (COVID-19) (Long COVID) | DRUG: RSLV-132\|DRUG: Sodium Chloride 0.9% |
| NCT05497089 | Temelimab as a Disease Modifying Therapy in Patients With Neuropsychiatric Symptoms in Post-COVID 19 or PASC Syndrome | https://clinicaltrials.gov/study/NCT05497089 | COMPLETED | Post-COVID-19 Syndrome | DRUG: Temelimab 54mg/kg\|DRUG: Placebo |
| NCT05682560 | Human Umbilical Cord Blood (RegeneCyte) Infusion in Patients with Post-COVID Syndrome | https://clinicaltrials.gov/study/NCT05682560 | COMPLETED | Long COVID\|Post-COVID Syndrome\|Post COVID-19 Condition | BIOLOGICAL: REGENECYTE\|BIOLOGICAL: Placebo |
| NCT03554265 | Brain and Gut Plasticity in Mild TBI or Post-acute COVID Syndrome Following Growth Hormone Therapy | https://clinicaltrials.gov/study/NCT03554265 | COMPLETED | Traumatic Brain Injury\|Fatigue\|Cognitive Impairment\|COVID-19 | DRUG: Somatropin |
| NCT05823896 | ImPROving Quality of LIFe in the Long COVID Patient | https://clinicaltrials.gov/study/NCT05823896 | COMPLETED | Post-COVID-19 Syndrome\|Long COVID\|Long Covid19\|COVID-19\|POTS - Postural Orthostatic Tachycardia Syndrome\|Post COVID-19 Condition\|Post-COVID Syndrome\|Post COVID-19 Condition, Unspecified\|Postinfectious Inflammation\|Postinfectious Disorder | DRUG: Nirmatrelvir/ritonavir\|DRUG: Placebo/ritonavir |
| NCT05679505 | Vagus Nerve Stimulation for Post-COVID Syndrome | https://clinicaltrials.gov/study/NCT05679505 | COMPLETED | Long COVID\|Vagus Nerve Stimulations\|Heart Rates\|Autonomic Nervous System Disorders | DEVICE: Auricular transcutaneous vagus nerve stimulation |
| NCT04880161 | A Study to Evaluate Ampion in Patients With Prolonged Respiratory Symptoms Due to COVID-19 (Long COVID) | https://clinicaltrials.gov/study/NCT04880161 | COMPLETED | Covid19 | BIOLOGICAL: Ampion\|OTHER: Placebo |
| NCT06383819 | Efficacy and Safety of Longidaza¬Æ for the Treatment of Patients With Residual Changes in the Lungs After COVID-19 | https://clinicaltrials.gov/study/NCT06383819 | COMPLETED | Post-Acute COVID-19 Syndrome\|Lung Disease With Polymyositis\|Fibrosis\|Lung Diseases, Interstitial\|Lung; Disease, Interstitial, With Fibrosis | DRUG: Longidaza¬Æ\|DRUG: Placebo |
| NCT05445674 | Plasma Exchange Therapy for Post- COVID-19 Condition: A Pilot, Randomized Double-Blind Study | https://clinicaltrials.gov/study/NCT05445674 | COMPLETED | Post-COVID19 Condition | COMBINATION_PRODUCT: Plasma Exchange Procedure\|OTHER: Sham Plasma Exchange Procedure |
| NCT05841498 | Immunoadsorption Study Mainz in Adults With Post-COVID Syndrome | https://clinicaltrials.gov/study/NCT05841498 | COMPLETED | Post-COVID-19 Syndrome\|Post-COVID Syndrome\|Post COVID-19 Condition | DEVICE: Immunoadsorption\|DEVICE: Sham-apheresis |
| NCT05764070 | Impact of Vagus Nerve Stimulation on Post-Aerobic Activity Recovery in Post SARS-CoV-2 Patients | https://clinicaltrials.gov/study/NCT05764070 | COMPLETED | Post-COVID-19 Syndrome | DEVICE: non-invasive auricular vagus stimulation\|DEVICE: Placebo Non Invasive Vagus Stimulation |
| NCT05126563 | Randomized Double-Blind Phase 2 Study of Allogeneic HB-adMSCs for the Treatment of Chronic Post-COVID-19 Syndrome | https://clinicaltrials.gov/study/NCT05126563 | COMPLETED | Post COVID-19 Syndrome | BIOLOGICAL: HB-adMSCs (allogeneic)\|OTHER: Placebo |
| NCT05633407 | Efficacy and Safety Study of Efgartigimod in Adults With Post-COVID-19 POTS | https://clinicaltrials.gov/study/NCT05633407 | COMPLETED | Postural Orthostatic Tachycardia Syndrome | DRUG: Efgartigimod\|DRUG: Placebo |
| NCT05630040 | VNS for Long-COVID-19 | https://clinicaltrials.gov/study/NCT05630040 | COMPLETED | Post-COVID-19 Syndrome\|Postural Tachycardia Syndrome\|Dysautonomia | DEVICE: Non-invasive vagus nerve stimulation\|DEVICE: Sham Intervention |
| NCT05225220 | Multimodal Investigation of Post COVID-19 in Females | https://clinicaltrials.gov/study/NCT05225220 | COMPLETED | Post COVID-19\|Cognitive Dysfunction | DEVICE: Parasym Device (of Parasym Ltd, UK) using Transcutaneous Vagus Nerve Stimulation (t-VNS) |
| NCT05638633 | Prednisolone and Vitamin B1/6/12 in Patients With Post-Covid-Syndrome | https://clinicaltrials.gov/study/NCT05638633 | COMPLETED | Post-COVID-19 Syndrome | DRUG: Prednisolone 20 mg/ 5 mg\|DRUG: Vitamin B compound (100mg B1, 50 mg B6, 500 ¬µg B12)\|DRUG: Placebo for Vitamin B compound\|DRUG: Placebo for Prednisolon |
| NCT04678830 | Double Blind, Placebo Controlled Study of Safety and Efficacy of Leronlimab in Patients With "Long" COVID-19 | https://clinicaltrials.gov/study/NCT04678830 | COMPLETED | Coronavirus Disease 2019 | DRUG: Placebos\|DRUG: Leronlimab (700mg) |
| NCT05228899 | Zofin to Treat COVID-19 Long Haulers | https://clinicaltrials.gov/study/NCT05228899 | COMPLETED | COVID-19 | DRUG: Zofin\|OTHER: Placebo |

**List of *terminated* Long COVID trials testing potentially disease modifying interventions**

| **NCT Number** | **Study Title** | **Study URL** | **Study Status** | **Conditions** | **Interventions** |
| --- | --- | --- | --- | --- | --- |
| NCT05918978 | Open Label Extension of Efgartigimod in Adults with Post-COVID-19 POTS | https://clinicaltrials.gov/study/NCT05918978 | TERMINATED | Post-COVID Postural Orthostatic Tachycardia Syndrome Postural Orthostatic Tachycardia Syndrome | DRUG: Efgartigimod |

**Appendix 3. Methods of rapid literature review on ethical aspects of Long COVID trials.**

| **Search date** | October 24, 2025 |
| --- | --- |
| **Database(s)** | Embase (including Medline and PubMed) |
| **Search string(s)** | ('long covid'/exp OR 'long covid' OR 'long covid19' OR 'long covid-19' OR 'post-acute covid' OR 'post-acute covid-19'/exp OR 'post-acute covid-19' OR 'post-acute covid19' OR 'post-acute sequelae of sars-cov-2 infection'/exp OR 'post-acute sequelae of sars-cov-2 infection' OR pasc OR 'post-acute sequelae of covid' OR 'post-acute sequelae of covid19' OR 'post-acute sequelae of covid-19' OR 'persistent covid-19' OR 'persistent COVID 19' OR 'long haul* covid' OR 'long-haul* covid-19' OR 'chronic covid' OR 'Post-COVID condition*' OR 'Post COVID condition*' OR 'Post-COVID-19 condition*' OR 'Post COVID-19 condition*' OR 'Post-COVID 19 condition*' OR 'Post COVID 19 condition*' OR 'PCC' OR 'Post-COVID syndrome*' OR 'Post COVID syndrome*' OR 'Post-COVID-19 syndrome*' OR 'Post COVID-19 syndrome*' OR 'Post-COVID 19 syndrome*' OR 'Post COVID 19 syndrome*' OR 'long-term effects of COVID' OR 'longterm effects of COVID') AND (ethic* OR moral* OR bioethic* OR unethic*)   - No search limits (e.g., publication date, language) |
|  | Rationale: We combined terms related to ethics and long Covid to identify existing literature on ethical aspects of Long COVID trials that could inform this paper. |
| **Selection criteria** | 1. Articles in academic journals AND 2. Consider how Long COVID trials should be conducted using a dedicated ethical perspective*. * Defined as making normative claims about trial design based on explicit ethical analysis or using established ethical frameworks (as opposed to making claims based on e.g., scientific considerations, patient-centeredness, etc.) |
|  | Rationale: We used these selection criteria to capture the full range of ethical perspectives on Long COVID treatment trials. |
| **Reviewer** | Primary: Saskia Hendriks, secondary (as needed): Annette Rid. |
| **Search design and summary of results** | Saskia Hendriks, Annette Rid. |

**Appendix 4. Results of rapid literature search on ethical aspects of Long COVID trials.**

Records identified from Embase (n = 1233)

Records removed *before screening*:

Records that were published ≤2019 removed (n= 455)

Duplicate records removed (n = 9)

**Identification**

**Included**

Articles sought for retrieval

(n = 42)

Reports not retrieved

(n = 0)

Articles assessed for eligibility

(n = 42)

Reports excluded: (n = 40)

Articles included in review

(n = 2) ^1,2^

Articles included based on review of references from included publications (n = 0)

Articles included based on personal databases of the authors (n = 0)

Records screened

(n = 769)

Records excluded based on title/abstract screening

(n = 727)

**Screening**

**Identification of studies via databases and registers**

Source of Flow Chart Template: ^3^

**References**

1. Smith EM, Anderson EE, Deer R, Prochaska J, Bohn K, Croisant S. Reviewing fair subject selection considerations for the unique case of post sequelae COVID-19 translational studies. *J Clin Transl Sci* 2022; **6**(1): e91.

2. Medeiros M, Edwards HA, Baquet CR. Research in the USA on COVID-19's long-term effects: measures needed to ensure black, indigenous and Latinx communities are not left behind. *J Med Ethics* 2023; **49**(2): 87–91.

3. Page MJ, McKenzie JE, Bossuyt PM, et al. The PRISMA 2020 statement: an updated guideline for reporting systematic reviews. *BMJ* 2021; **372**: n71.
